# *N*,*N*-Diethyl-4-[9-meth­oxy-6-(4-methoxy­phen­yl)-5-methyl-2-phenyl-2*H*-benzo[*h*]chromen-2-yl]aniline

**DOI:** 10.1107/S1600536811011925

**Published:** 2011-04-07

**Authors:** Moon-Hwan Kim, Hee-Moon Park, Chong-Hyeak Kim

**Affiliations:** a Biomaterial Research Center, Korea Research Institute of Chemical Technology, PO Box 107, Yuseong, Daejeon 305-600, Republic of Korea; b Environment and Resources Research Center, Korea Research Institute of Chemical Technology, PO Box 107, Yuseong, Daejeon 305-600, Republic of Korea; c Center for Chemical Analysis, Korea Research Institute of Chemical Technology, PO Box 107, Yuseong, Daejeon 305-600, Republic of Korea

## Abstract

In the title compound, C_38_H_37_NO_3_, the pyran ring has an envelope conformation with the quaternary C_q_ atom as the flap atom. The dihedral angle formed between the meth­oxy­phenyl group and the naphthalene ring system is 67.32 (6)°. The ethyl­amino groups lie to the same side of the plane through the phenyl ring and form dihedral angles of 84.6 (3) and 75.8 (2)° with it.

## Related literature

For the synthesis and structures of photochromic benzo- and naphtho­pyrans, see: Kim *et al.* (2010 ▶, 2011 ▶); Do *et al.* (2011 ▶). For the synthesis and applications of organic photochromic and thermochromic dyes, see: Kumar *et al.* (1995 ▶); Crano & Guglielmetti (1999 ▶); Gabbutt *et al.* (2003 ▶, 2004 ▶); Gemert & Selvig (2000 ▶); Nelson *et al.* (2002 ▶).
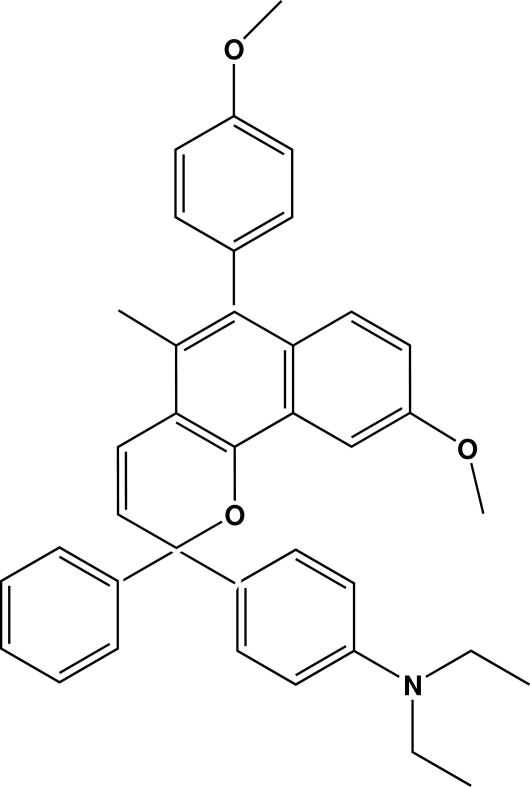


## Experimental

### 

#### Crystal data

C_38_H_37_NO_3_
*M* *_r_* = 555.69
Triclinic, 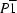

*a* = 11.0527 (3) Å
*b* = 11.6870 (3) Å
*c* = 12.2752 (3) Å
α = 102.104 (2)°
β = 93.012 (2)°
γ = 95.929 (2)°
*V* = 1537.58 (7) Å^3^
*Z* = 2
Mo *K*α radiation
μ = 0.08 mm^−1^
*T* = 296 K
0.20 × 0.18 × 0.07 mm

#### Data collection

Bruker APEXII CCD diffractometer
28232 measured reflections
7658 independent reflections
2360 reflections with *I* > 2σ(*I*)
*R*_int_ = 0.091

#### Refinement

*R*[*F* ^2^ > 2σ(*F* ^2^)] = 0.064
*wR*(*F* ^2^) = 0.181
*S* = 0.92
7658 reflections
381 parameters
H-atom parameters constrained
Δρ_max_ = 0.17 e Å^−3^
Δρ_min_ = −0.16 e Å^−3^

### 

Data collection: *APEX2* (Bruker, 2009 ▶); cell refinement: *SAINT* (Bruker, 2009 ▶); data reduction: *SAINT*; program(s) used to solve structure: *SHELXS97* (Sheldrick, 2008 ▶); program(s) used to refine structure: *SHELXL97* (Sheldrick, 2008 ▶); molecular graphics: *XP* in *SHELXTL* (Sheldrick, 2008 ▶); software used to prepare material for publication: *SHELXL97*.

## Supplementary Material

Crystal structure: contains datablocks global, I. DOI: 10.1107/S1600536811011925/tk2732sup1.cif

Structure factors: contains datablocks I. DOI: 10.1107/S1600536811011925/tk2732Isup2.hkl

Additional supplementary materials:  crystallographic information; 3D view; checkCIF report

## Figures and Tables

**Fig. 1.**
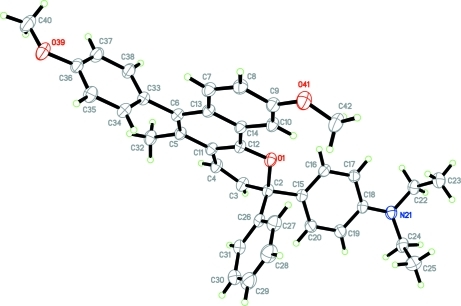
A view of molecule of the title compound showing the atom numbering scheme. Displacement ellipsoids are drawn at the 25% probability level. H atoms are presented as a small spheres of arbitrary radius.

## supplementary crystallographic information

### Comment

The synthesis and applications of the organic photochromic and thermochromic dyes has become of great interest (Kumar *et al.*, 1995; Gemert & Selvig, 2000; Nelson *et al.*, 2002;  Gabbutt *et al.*, 2003, 2004). For example, they may be useful as optical transmission materials for ophthalmic glasses and lenses. They also have potential use for optical storage of optical disks or memories (Crano & Guglielmetti, 1999). In particular, benzo- and naphtho-pyrans have been commercialized as photochromic plastic sunglasses in the early 1990's. In our group, research has focused on the development of novel photochromic benzo- and naphtho-pyrans (Kim *et al.*, 2010, 2011; Do *et al.*, 2011). Herein, we report the crystal structure of *N*,*N*-diethyl-4-(9-methoxy-6-(4-methoxyphenyl)-5-methyl-2-phenyl-2*H*- benzo[*h*]chromene-2-yl)benzenamine (Fig. 1) as a new photochromic material. The –C2—O1—C12—C11—C4—C3- pyran ring has an envelope conformation with the quaternary C2 atom out of the plane, the dihedral angle is 38.7 (2)°, C2—O1 is 1.456 (3) Å and C2—C3 is 1.506 (3) Å. The dihedral angle formed between the methoxyphenyl group and the naphthalen ring of the naphthopyran substituent is 67.32 (6) °. The ethylamino groups of the diethylaminophenyl substituent form dihedral angles of 75.8 (2) and 84.6 (3) ° with the plane through the phenyl ring.

### Experimental

A solution of 7-methoxy-4-(4-methoxyphenyl)-3-methylnaphthalen-1-ol (10 g, 0.04 mol) and 1-(4-diethylaminophenyl)-1-phenylprop-2-yn-1-ol (11.18 g, 0.04 mol) in anhydrous toluene (500 ml) containing acidic alumina (32.6 g, 0.32 mol) was refluxed for 4 h. The cooled resulting solution was filtered and the alumina residue washed well with toluene. The organic layer was washed with aqueous NaOH and extracted with ethyl acetate (350 ml) and washed with water. The extracts were dried with anhydrous MgSO_4_ and evaporated to give a crude orange oil. This was purified by silica gel column chromatograpy using 10% ethyl acetate/15% toluene in hexane as eluent. The purified yellow oil was solidified on trituration with ethyl ether. The solid obtained was suction filtered, washed with ethyl ether and air dried (yield: 6.2 g, 28%). Single crystals were obtained by recrystallization from its ethyl acetate solution.

### Refinement

Carbon-bound H-atoms were placed in their calculated positions (C-H = 0.93 to 0.97 Å) and were included in the refinement in the riding model approximation with *U_iso_*(H) = 1.2*U_eq_*(C) and 1.5*U*_eq_(methyl-C).

### Crystal data

**Table:**

C_38_H_37_NO_3_	*Z* = 2
*M**_r_* = 555.69	*F*(000) = 592
Triclinic, *P*1	*D*_x_ = 1.200 Mg m^−^^3^
Hall symbol: -P 1	Mo *K*α radiation, λ = 0.71073 Å
*a* = 11.0527 (3) Å	Cell parameters from 1662 reflections
*b* = 11.6870 (3) Å	θ = 2.4–18.2°
*c* = 12.2752 (3) Å	µ = 0.08 mm^−^^1^
α = 102.104 (2)°	*T* = 296 K
β = 93.012 (2)°	Block, silver
γ = 95.929 (2)°	0.20 × 0.18 × 0.07 mm
*V* = 1537.58 (7) Å^3^	

### Data collection

**Table:**

Bruker APEXII CCD diffractometer	2360 reflections with *I* > 2σ(*I*)
Radiation source: fine-focus sealed tube	*R*_int_ = 0.091
graphite	θ_max_ = 28.5°, θ_min_ = 1.7°
φ and ω scans	*h* = −11→14
28232 measured reflections	*k* = −11→15
7658 independent reflections	*l* = −16→16

### Refinement

**Table:**

Refinement on *F*^2^	Secondary atom site location: difference Fourier map
Least-squares matrix: full	Hydrogen site location: inferred from neighbouring sites
*R*[*F*^2^ > 2σ(*F*^2^)] = 0.064	H-atom parameters constrained
*wR*(*F*^2^) = 0.181	*w* = 1/[σ^2^(*F*_o_^2^) + (0.0678*P*)^2^] where *P* = (*F*_o_^2^ + 2*F*_c_^2^)/3
*S* = 0.92	(Δ/σ)_max_ < 0.001
7658 reflections	Δρ_max_ = 0.17 e Å^−^^3^
381 parameters	Δρ_min_ = −0.16 e Å^−^^3^
0 restraints	Extinction correction: *SHELXL*, Fc^*^=kFc[1+0.001xFc^2^λ^3^/sin(2θ)]^-1/4^
Primary atom site location: structure-invariant direct methods	Extinction coefficient: 0.0110 (16)

### Special details

**Table:**

Geometry. All e.s.d.'s (except the e.s.d. in the dihedral angle between two l.s. planes) are estimated using the full covariance matrix. The cell e.s.d.'s are taken into account individually in the estimation of e.s.d.'s in distances, angles and torsion angles; correlations between e.s.d.'s in cell parameters are only used when they are defined by crystal symmetry. An approximate (isotropic) treatment of cell e.s.d.'s is used for estimating e.s.d.'s involving l.s. planes.
Refinement. Refinement of *F*^2^ against ALL reflections. The weighted *R*-factor *wR* and goodness of fit *S* are based on *F*^2^, conventional *R*-factors *R* are based on *F*, with *F* set to zero for negative *F*^2^. The threshold expression of *F*^2^ > σ(*F*^2^) is used only for calculating *R*-factors(gt) *etc*. and is not relevant to the choice of reflections for refinement. *R*-factors based on *F*^2^ are statistically about twice as large as those based on *F*, and *R*- factors based on ALL data will be even larger.

### Fractional atomic coordinates and isotropic or equivalent isotropic displacement parameters (Å^2^)

**Table:**

	*x*	*y*	*z*	*U*_iso_*/*U*_eq_	
O1	0.82318 (17)	0.35611 (16)	0.23249 (14)	0.0615 (6)	
C2	0.9480 (3)	0.3713 (2)	0.2008 (2)	0.0554 (8)	
C3	0.9498 (3)	0.3032 (3)	0.0823 (2)	0.0661 (9)	
H3A	1.0016	0.3315	0.0342	0.079*	
C4	0.8776 (3)	0.2030 (3)	0.0465 (2)	0.0645 (8)	
H4A	0.8814	0.1596	−0.0257	0.077*	
C5	0.7291 (3)	0.0441 (2)	0.0983 (2)	0.0572 (8)	
C6	0.6450 (3)	0.0136 (2)	0.1700 (2)	0.0558 (8)	
C7	0.5308 (3)	0.0780 (3)	0.3385 (2)	0.0661 (9)	
H7A	0.4857	0.0040	0.3253	0.079*	
C8	0.5107 (3)	0.1610 (3)	0.4293 (3)	0.0701 (9)	
H8A	0.4534	0.1426	0.4776	0.084*	
C9	0.5755 (3)	0.2747 (3)	0.4509 (2)	0.0641 (8)	
C10	0.6585 (3)	0.3025 (2)	0.3801 (2)	0.0613 (8)	
H10A	0.7005	0.3779	0.3935	0.074*	
C11	0.7918 (3)	0.1603 (3)	0.1200 (2)	0.0561 (8)	
C12	0.7684 (3)	0.2420 (2)	0.2116 (2)	0.0557 (8)	
C13	0.6185 (2)	0.1013 (2)	0.2638 (2)	0.0560 (8)	
C14	0.6808 (2)	0.2169 (2)	0.2862 (2)	0.0554 (8)	
C15	0.9789 (3)	0.5028 (3)	0.2099 (2)	0.0553 (8)	
C16	0.8919 (3)	0.5786 (3)	0.2070 (2)	0.0631 (8)	
H16A	0.8099	0.5486	0.1987	0.076*	
C17	0.9231 (3)	0.6986 (3)	0.2159 (2)	0.0621 (8)	
H17A	0.8617	0.7468	0.2131	0.075*	
C18	1.0442 (3)	0.7479 (3)	0.2291 (2)	0.0567 (8)	
C19	1.1317 (3)	0.6707 (3)	0.2304 (3)	0.0724 (9)	
H19A	1.2139	0.6998	0.2379	0.087*	
C20	1.0988 (3)	0.5522 (3)	0.2210 (2)	0.0701 (9)	
H20A	1.1599	0.5034	0.2221	0.084*	
N21	1.0794 (2)	0.8671 (2)	0.23776 (19)	0.0649 (7)	
C22	0.9879 (3)	0.9483 (3)	0.2335 (2)	0.0752 (9)	
H22A	0.9214	0.9084	0.1798	0.090*	
H22B	1.0244	1.0148	0.2060	0.090*	
C23	0.9360 (3)	0.9942 (3)	0.3426 (3)	0.0904 (11)	
H23A	0.8768	1.0461	0.3316	0.136*	
H23B	1.0004	1.0365	0.3959	0.136*	
H23C	0.8977	0.9294	0.3700	0.136*	
C24	1.2021 (3)	0.9184 (3)	0.2814 (3)	0.0767 (10)	
H24A	1.2136	0.9992	0.2723	0.092*	
H24B	1.2599	0.8756	0.2372	0.092*	
C25	1.2312 (3)	0.9176 (3)	0.4035 (3)	0.0969 (11)	
H25A	1.3135	0.9528	0.4261	0.145*	
H25B	1.2224	0.8379	0.4132	0.145*	
H25C	1.1760	0.9617	0.4484	0.145*	
C26	1.0304 (3)	0.3294 (2)	0.2838 (3)	0.0601 (8)	
C27	1.0090 (3)	0.3521 (3)	0.3961 (3)	0.0795 (10)	
H27A	0.9399	0.3868	0.4189	0.095*	
C28	1.0877 (5)	0.3243 (4)	0.4747 (3)	0.1048 (13)	
H28A	1.0709	0.3393	0.5494	0.126*	
C29	1.1913 (5)	0.2744 (3)	0.4426 (4)	0.1074 (14)	
H29A	1.2446	0.2558	0.4958	0.129*	
C30	1.2159 (4)	0.2522 (3)	0.3323 (4)	0.0959 (12)	
H30A	1.2869	0.2204	0.3108	0.115*	
C31	1.1345 (3)	0.2773 (3)	0.2529 (3)	0.0772 (10)	
H31A	1.1498	0.2589	0.1779	0.093*	
C32	0.7596 (3)	−0.0455 (3)	−0.0027 (2)	0.0748 (9)	
H32A	0.7356	−0.1237	0.0074	0.112*	
H32B	0.8458	−0.0354	−0.0104	0.112*	
H32C	0.7166	−0.0339	−0.0687	0.112*	
C33	0.5811 (3)	−0.1077 (3)	0.1530 (2)	0.0569 (8)	
C34	0.6030 (3)	−0.1781 (3)	0.2284 (2)	0.0653 (9)	
H34A	0.6588	−0.1485	0.2898	0.078*	
C35	0.5437 (3)	−0.2912 (3)	0.2146 (3)	0.0720 (9)	
H35A	0.5607	−0.3369	0.2658	0.086*	
C36	0.4591 (3)	−0.3361 (3)	0.1245 (3)	0.0649 (9)	
C37	0.4360 (3)	−0.2686 (3)	0.0496 (3)	0.0766 (10)	
H37A	0.3796	−0.2981	−0.0114	0.092*	
C38	0.4964 (3)	−0.1562 (3)	0.0642 (3)	0.0740 (9)	
H38A	0.4794	−0.1114	0.0122	0.089*	
O39	0.4055 (2)	−0.44842 (19)	0.11944 (19)	0.0864 (7)	
C40	0.3228 (3)	−0.5006 (3)	0.0241 (3)	0.0993 (12)	
H40A	0.2913	−0.5788	0.0294	0.149*	
H40B	0.3651	−0.5043	−0.0426	0.149*	
H40C	0.2567	−0.4538	0.0218	0.149*	
O41	0.54847 (19)	0.34888 (19)	0.54643 (16)	0.0835 (7)	
C42	0.6237 (3)	0.4591 (3)	0.5807 (3)	0.0965 (12)	
H42A	0.5959	0.5033	0.6478	0.145*	
H42B	0.6190	0.5027	0.5228	0.145*	
H42C	0.7066	0.4452	0.5945	0.145*	

### Atomic displacement parameters (Å^2^)

**Table:**

	*U*^11^	*U*^22^	*U*^33^	*U*^12^	*U*^13^	*U*^23^
O1	0.0663 (14)	0.0527 (13)	0.0626 (12)	−0.0051 (11)	0.0187 (10)	0.0084 (10)
C2	0.059 (2)	0.052 (2)	0.0522 (17)	−0.0041 (16)	0.0157 (15)	0.0075 (14)
C3	0.074 (2)	0.065 (2)	0.0551 (18)	−0.0070 (18)	0.0164 (16)	0.0067 (16)
C4	0.078 (2)	0.061 (2)	0.0502 (17)	−0.0023 (18)	0.0116 (16)	0.0049 (15)
C5	0.063 (2)	0.056 (2)	0.0482 (17)	0.0010 (16)	0.0037 (15)	0.0042 (14)
C6	0.0562 (19)	0.053 (2)	0.0548 (17)	−0.0035 (15)	0.0010 (15)	0.0104 (15)
C7	0.062 (2)	0.063 (2)	0.069 (2)	−0.0065 (16)	0.0144 (17)	0.0091 (17)
C8	0.064 (2)	0.068 (2)	0.078 (2)	−0.0015 (18)	0.0227 (17)	0.0151 (18)
C9	0.065 (2)	0.064 (2)	0.0604 (19)	0.0020 (18)	0.0162 (17)	0.0058 (16)
C10	0.066 (2)	0.055 (2)	0.0598 (18)	−0.0054 (16)	0.0141 (16)	0.0089 (16)
C11	0.059 (2)	0.055 (2)	0.0516 (17)	−0.0028 (16)	0.0062 (15)	0.0105 (15)
C12	0.063 (2)	0.0472 (19)	0.0537 (17)	−0.0017 (16)	0.0024 (15)	0.0089 (15)
C13	0.056 (2)	0.056 (2)	0.0544 (17)	−0.0016 (16)	0.0055 (15)	0.0133 (15)
C14	0.0537 (19)	0.053 (2)	0.0553 (17)	−0.0033 (15)	0.0076 (15)	0.0070 (15)
C15	0.061 (2)	0.052 (2)	0.0531 (17)	0.0017 (18)	0.0108 (15)	0.0109 (14)
C16	0.060 (2)	0.062 (2)	0.0596 (18)	−0.0054 (19)	0.0040 (15)	0.0025 (15)
C17	0.066 (2)	0.057 (2)	0.0609 (19)	0.0090 (18)	0.0035 (16)	0.0087 (15)
C18	0.067 (2)	0.047 (2)	0.0551 (17)	−0.0009 (19)	0.0166 (15)	0.0112 (14)
C19	0.062 (2)	0.056 (2)	0.100 (2)	0.0016 (19)	0.0219 (18)	0.0188 (18)
C20	0.060 (2)	0.055 (2)	0.096 (2)	0.0036 (18)	0.0186 (18)	0.0155 (17)
N21	0.073 (2)	0.0515 (17)	0.0709 (16)	0.0042 (16)	0.0119 (14)	0.0152 (13)
C22	0.095 (3)	0.058 (2)	0.075 (2)	0.007 (2)	0.0074 (19)	0.0198 (17)
C23	0.098 (3)	0.085 (3)	0.083 (2)	0.017 (2)	0.011 (2)	0.0035 (19)
C24	0.085 (3)	0.056 (2)	0.089 (3)	−0.0055 (18)	0.018 (2)	0.0171 (18)
C25	0.085 (3)	0.104 (3)	0.098 (3)	−0.003 (2)	0.003 (2)	0.022 (2)
C26	0.068 (2)	0.0423 (18)	0.065 (2)	−0.0039 (16)	0.0014 (17)	0.0066 (15)
C27	0.097 (3)	0.076 (2)	0.064 (2)	0.005 (2)	0.000 (2)	0.0158 (19)
C28	0.134 (4)	0.095 (3)	0.081 (3)	0.003 (3)	−0.011 (3)	0.020 (2)
C29	0.132 (4)	0.072 (3)	0.114 (4)	−0.004 (3)	−0.037 (3)	0.030 (3)
C30	0.097 (3)	0.059 (2)	0.126 (4)	0.009 (2)	−0.018 (3)	0.014 (2)
C31	0.083 (3)	0.054 (2)	0.090 (2)	0.0064 (19)	0.003 (2)	0.0066 (18)
C32	0.092 (2)	0.067 (2)	0.0597 (18)	0.0008 (18)	0.0116 (17)	0.0022 (16)
C33	0.059 (2)	0.052 (2)	0.0575 (18)	−0.0002 (16)	0.0043 (15)	0.0108 (16)
C34	0.066 (2)	0.063 (2)	0.0611 (19)	−0.0094 (17)	0.0018 (15)	0.0092 (17)
C35	0.084 (2)	0.065 (2)	0.067 (2)	−0.0055 (19)	0.0119 (18)	0.0188 (17)
C36	0.063 (2)	0.054 (2)	0.073 (2)	−0.0048 (18)	0.0151 (18)	0.0062 (18)
C37	0.080 (2)	0.065 (2)	0.076 (2)	−0.007 (2)	−0.0109 (18)	0.0072 (19)
C38	0.079 (2)	0.059 (2)	0.078 (2)	−0.0061 (18)	−0.0090 (18)	0.0115 (17)
O39	0.0922 (17)	0.0628 (16)	0.0942 (16)	−0.0195 (13)	0.0114 (13)	0.0068 (12)
C40	0.093 (3)	0.077 (3)	0.107 (3)	−0.024 (2)	0.010 (2)	−0.012 (2)
O41	0.0940 (17)	0.0751 (16)	0.0749 (14)	−0.0036 (13)	0.0343 (12)	0.0015 (12)
C42	0.108 (3)	0.078 (3)	0.086 (2)	−0.019 (2)	0.023 (2)	−0.0117 (19)

### Geometric parameters (Å, °)

**Table:**

O1—C12	1.373 (3)	C23—H23A	0.9600
O1—C2	1.456 (3)	C23—H23B	0.9600
C2—C3	1.506 (3)	C23—H23C	0.9600
C2—C15	1.518 (4)	C24—C25	1.518 (4)
C2—C26	1.521 (4)	C24—H24A	0.9700
C3—C4	1.325 (3)	C24—H24B	0.9700
C3—H3A	0.9300	C25—H25A	0.9600
C4—C11	1.464 (4)	C25—H25B	0.9600
C4—H4A	0.9300	C25—H25C	0.9600
C5—C6	1.383 (3)	C26—C27	1.385 (4)
C5—C11	1.424 (4)	C26—C31	1.390 (4)
C5—C32	1.523 (3)	C27—C28	1.376 (5)
C6—C13	1.437 (3)	C27—H27A	0.9300
C6—C33	1.486 (4)	C28—C29	1.377 (5)
C7—C8	1.360 (4)	C28—H28A	0.9300
C7—C13	1.414 (3)	C29—C30	1.371 (5)
C7—H7A	0.9300	C29—H29A	0.9300
C8—C9	1.409 (4)	C30—C31	1.389 (4)
C8—H8A	0.9300	C30—H30A	0.9300
C9—C10	1.357 (3)	C31—H31A	0.9300
C9—O41	1.371 (3)	C32—H32A	0.9600
C10—C14	1.410 (3)	C32—H32B	0.9600
C10—H10A	0.9300	C32—H32C	0.9600
C11—C12	1.369 (3)	C33—C38	1.382 (4)
C12—C14	1.418 (3)	C33—C34	1.388 (4)
C13—C14	1.416 (3)	C34—C35	1.386 (4)
C15—C20	1.377 (4)	C34—H34A	0.9300
C15—C16	1.377 (4)	C35—C36	1.386 (4)
C16—C17	1.388 (4)	C35—H35A	0.9300
C16—H16A	0.9300	C36—C37	1.362 (4)
C17—C18	1.389 (4)	C36—O39	1.371 (3)
C17—H17A	0.9300	C37—C38	1.383 (4)
C18—N21	1.388 (3)	C37—H37A	0.9300
C18—C19	1.390 (4)	C38—H38A	0.9300
C19—C20	1.374 (4)	O39—C40	1.431 (3)
C19—H19A	0.9300	C40—H40A	0.9600
C20—H20A	0.9300	C40—H40B	0.9600
N21—C24	1.452 (4)	C40—H40C	0.9600
N21—C22	1.462 (3)	O41—C42	1.429 (3)
C22—C23	1.501 (4)	C42—H42A	0.9600
C22—H22A	0.9700	C42—H42B	0.9600
C22—H22B	0.9700	C42—H42C	0.9600
			
C12—O1—C2	115.6 (2)	H23A—C23—H23B	109.5
O1—C2—C3	107.5 (2)	C22—C23—H23C	109.5
O1—C2—C15	105.6 (2)	H23A—C23—H23C	109.5
C3—C2—C15	111.6 (2)	H23B—C23—H23C	109.5
O1—C2—C26	108.2 (2)	N21—C24—C25	114.4 (2)
C3—C2—C26	113.4 (2)	N21—C24—H24A	108.7
C15—C2—C26	110.1 (2)	C25—C24—H24A	108.7
C4—C3—C2	120.1 (3)	N21—C24—H24B	108.7
C4—C3—H3A	119.9	C25—C24—H24B	108.7
C2—C3—H3A	119.9	H24A—C24—H24B	107.6
C3—C4—C11	120.3 (3)	C24—C25—H25A	109.5
C3—C4—H4A	119.8	C24—C25—H25B	109.5
C11—C4—H4A	119.8	H25A—C25—H25B	109.5
C6—C5—C11	120.1 (2)	C24—C25—H25C	109.5
C6—C5—C32	121.3 (3)	H25A—C25—H25C	109.5
C11—C5—C32	118.6 (3)	H25B—C25—H25C	109.5
C5—C6—C13	119.3 (3)	C27—C26—C31	117.6 (3)
C5—C6—C33	121.9 (3)	C27—C26—C2	120.0 (3)
C13—C6—C33	118.8 (2)	C31—C26—C2	122.1 (3)
C8—C7—C13	121.6 (3)	C28—C27—C26	121.5 (4)
C8—C7—H7A	119.2	C28—C27—H27A	119.3
C13—C7—H7A	119.2	C26—C27—H27A	119.3
C7—C8—C9	120.9 (3)	C27—C28—C29	120.0 (4)
C7—C8—H8A	119.6	C27—C28—H28A	120.0
C9—C8—H8A	119.6	C29—C28—H28A	120.0
C10—C9—O41	125.3 (3)	C30—C29—C28	120.0 (4)
C10—C9—C8	119.9 (3)	C30—C29—H29A	120.0
O41—C9—C8	114.8 (3)	C28—C29—H29A	120.0
C9—C10—C14	119.8 (3)	C29—C30—C31	119.8 (4)
C9—C10—H10A	120.1	C29—C30—H30A	120.1
C14—C10—H10A	120.1	C31—C30—H30A	120.1
C12—C11—C5	119.9 (3)	C30—C31—C26	121.1 (3)
C12—C11—C4	115.7 (3)	C30—C31—H31A	119.5
C5—C11—C4	124.3 (3)	C26—C31—H31A	119.5
C11—C12—O1	121.5 (3)	C5—C32—H32A	109.5
C11—C12—C14	122.5 (3)	C5—C32—H32B	109.5
O1—C12—C14	115.8 (2)	H32A—C32—H32B	109.5
C7—C13—C14	116.4 (3)	C5—C32—H32C	109.5
C7—C13—C6	122.7 (3)	H32A—C32—H32C	109.5
C14—C13—C6	120.9 (3)	H32B—C32—H32C	109.5
C10—C14—C13	121.4 (3)	C38—C33—C34	116.4 (3)
C10—C14—C12	121.4 (3)	C38—C33—C6	122.8 (3)
C13—C14—C12	117.2 (3)	C34—C33—C6	120.7 (3)
C20—C15—C16	116.4 (3)	C35—C34—C33	121.7 (3)
C20—C15—C2	120.4 (3)	C35—C34—H34A	119.2
C16—C15—C2	123.3 (3)	C33—C34—H34A	119.2
C15—C16—C17	121.9 (3)	C36—C35—C34	120.0 (3)
C15—C16—H16A	119.0	C36—C35—H35A	120.0
C17—C16—H16A	119.0	C34—C35—H35A	120.0
C16—C17—C18	121.3 (3)	C37—C36—O39	125.6 (3)
C16—C17—H17A	119.4	C37—C36—C35	119.4 (3)
C18—C17—H17A	119.4	O39—C36—C35	115.0 (3)
N21—C18—C17	123.1 (3)	C36—C37—C38	119.9 (3)
N21—C18—C19	120.3 (3)	C36—C37—H37A	120.0
C17—C18—C19	116.6 (3)	C38—C37—H37A	120.0
C20—C19—C18	121.2 (3)	C33—C38—C37	122.6 (3)
C20—C19—H19A	119.4	C33—C38—H38A	118.7
C18—C19—H19A	119.4	C37—C38—H38A	118.7
C19—C20—C15	122.7 (3)	C36—O39—C40	116.8 (3)
C19—C20—H20A	118.7	O39—C40—H40A	109.5
C15—C20—H20A	118.7	O39—C40—H40B	109.5
C18—N21—C24	120.1 (3)	H40A—C40—H40B	109.5
C18—N21—C22	120.6 (3)	O39—C40—H40C	109.5
C24—N21—C22	117.2 (2)	H40A—C40—H40C	109.5
N21—C22—C23	115.4 (3)	H40B—C40—H40C	109.5
N21—C22—H22A	108.4	C9—O41—C42	117.0 (2)
C23—C22—H22A	108.4	O41—C42—H42A	109.5
N21—C22—H22B	108.4	O41—C42—H42B	109.5
C23—C22—H22B	108.4	H42A—C42—H42B	109.5
H22A—C22—H22B	107.5	O41—C42—H42C	109.5
C22—C23—H23A	109.5	H42A—C42—H42C	109.5
C22—C23—H23B	109.5	H42B—C42—H42C	109.5
			
C12—O1—C2—C3	49.7 (3)	C20—C15—C16—C17	−0.8 (4)
C12—O1—C2—C15	169.0 (2)	C2—C15—C16—C17	179.8 (2)
C12—O1—C2—C26	−73.1 (3)	C15—C16—C17—C18	−0.4 (4)
O1—C2—C3—C4	−34.5 (4)	C16—C17—C18—N21	179.6 (2)
C15—C2—C3—C4	−149.9 (3)	C16—C17—C18—C19	1.2 (4)
C26—C2—C3—C4	85.0 (3)	N21—C18—C19—C20	−179.5 (3)
C2—C3—C4—C11	2.7 (4)	C17—C18—C19—C20	−1.0 (4)
C11—C5—C6—C13	2.3 (4)	C18—C19—C20—C15	−0.1 (5)
C32—C5—C6—C13	−179.6 (3)	C16—C15—C20—C19	1.0 (4)
C11—C5—C6—C33	−177.6 (3)	C2—C15—C20—C19	−179.5 (3)
C32—C5—C6—C33	0.5 (4)	C17—C18—N21—C24	163.0 (3)
C13—C7—C8—C9	1.1 (5)	C19—C18—N21—C24	−18.6 (4)
C7—C8—C9—C10	0.6 (5)	C17—C18—N21—C22	0.1 (4)
C7—C8—C9—O41	−178.6 (3)	C19—C18—N21—C22	178.4 (3)
O41—C9—C10—C14	178.0 (3)	C18—N21—C22—C23	84.6 (3)
C8—C9—C10—C14	−1.0 (4)	C24—N21—C22—C23	−78.8 (3)
C6—C5—C11—C12	−0.3 (4)	C18—N21—C24—C25	−65.8 (4)
C32—C5—C11—C12	−178.5 (3)	C22—N21—C24—C25	97.8 (3)
C6—C5—C11—C4	−176.7 (3)	O1—C2—C26—C27	−38.5 (3)
C32—C5—C11—C4	5.1 (4)	C3—C2—C26—C27	−157.6 (3)
C3—C4—C11—C12	16.8 (4)	C15—C2—C26—C27	76.5 (3)
C3—C4—C11—C5	−166.6 (3)	O1—C2—C26—C31	147.3 (2)
C5—C11—C12—O1	−177.2 (2)	C3—C2—C26—C31	28.2 (4)
C4—C11—C12—O1	−0.5 (4)	C15—C2—C26—C31	−97.7 (3)
C5—C11—C12—C14	−1.5 (4)	C31—C26—C27—C28	0.1 (5)
C4—C11—C12—C14	175.2 (3)	C2—C26—C27—C28	−174.4 (3)
C2—O1—C12—C11	−34.8 (4)	C26—C27—C28—C29	0.9 (5)
C2—O1—C12—C14	149.2 (2)	C27—C28—C29—C30	−0.1 (6)
C8—C7—C13—C14	−2.3 (4)	C28—C29—C30—C31	−1.7 (6)
C8—C7—C13—C6	177.3 (3)	C29—C30—C31—C26	2.7 (5)
C5—C6—C13—C7	177.8 (3)	C27—C26—C31—C30	−1.9 (4)
C33—C6—C13—C7	−2.3 (4)	C2—C26—C31—C30	172.4 (3)
C5—C6—C13—C14	−2.6 (4)	C5—C6—C33—C38	−68.0 (4)
C33—C6—C13—C14	177.3 (3)	C13—C6—C33—C38	112.1 (3)
C9—C10—C14—C13	−0.2 (4)	C5—C6—C33—C34	113.1 (3)
C9—C10—C14—C12	−178.8 (3)	C13—C6—C33—C34	−66.8 (4)
C7—C13—C14—C10	1.8 (4)	C38—C33—C34—C35	0.6 (4)
C6—C13—C14—C10	−177.8 (3)	C6—C33—C34—C35	179.5 (3)
C7—C13—C14—C12	−179.5 (3)	C33—C34—C35—C36	−0.9 (4)
C6—C13—C14—C12	0.8 (4)	C34—C35—C36—C37	0.7 (4)
C11—C12—C14—C10	179.9 (3)	C34—C35—C36—O39	−179.6 (3)
O1—C12—C14—C10	−4.2 (4)	O39—C36—C37—C38	−180.0 (3)
C11—C12—C14—C13	1.2 (4)	C35—C36—C37—C38	−0.3 (4)
O1—C12—C14—C13	177.1 (2)	C34—C33—C38—C37	−0.2 (4)
O1—C2—C15—C20	159.1 (2)	C6—C33—C38—C37	−179.1 (3)
C3—C2—C15—C20	−84.4 (3)	C36—C37—C38—C33	0.1 (5)
C26—C2—C15—C20	42.5 (3)	C37—C36—O39—C40	3.2 (4)
O1—C2—C15—C16	−21.5 (3)	C35—C36—O39—C40	−176.4 (3)
C3—C2—C15—C16	95.1 (3)	C10—C9—O41—C42	−7.8 (4)
C26—C2—C15—C16	−138.1 (3)	C8—C9—O41—C42	171.3 (3)
